# Changes in opioid agonist treatment initiation among people prescribed opioids for pain following voluntary and mandatory prescription drug monitoring program implementation: A time series analysis

**DOI:** 10.1111/dar.13754

**Published:** 2023-10-05

**Authors:** Louisa Picco, Ting Xia, J. Simon Bell, Christopher Pearce, Rachelle Buchbinder, Dan I. Lubman, Suzanne Nielsen

**Affiliations:** ^1^ Monash Addiction Research Centre, Eastern Health Clinical School Monash University Melbourne Australia; ^2^ Centre for Medicine Use and Safety, Faculty of Pharmacy and Pharmaceutical Sciences Monash University Melbourne Australia; ^3^ School of Public Health and Preventive Medicine Monash University Melbourne Australia; ^4^ Outcome Health Melbourne Australia; ^5^ Turning Point, Eastern Health Clinical School Monash University Melbourne Australia

**Keywords:** buprenorphine, methadone, opioid agonist treatment, prescription drug monitoring program, real‐time prescription monitoring

## Abstract

**Introduction:**

Prescription drug monitoring programs (PDMP) are increasingly used to identify people prescribed high‐dose opioids. However, little is known about whether PDMPs impact opioid agonist treatment (OAT) uptake, the gold standard for opioid use disorder. This study examined the impact of PDMP implementation on OAT initiation among people prescribed opioids, in Victoria, Australia.

**Methods:**

De‐identified electronic records from all 464 Victorian general practices included in the POLAR database were used. OAT initiation was defined as a new OAT prescription between 1 April 2017 and 31 December 2020, with no OAT prescriptions in the year prior. Interrupted time series analyses were used to compare outcomes before (April 2017 to March 2019) and after (April 2019 to December 2020) PDMP implementation. Binary logistic regression was used to examine differences in patients' characteristics associated with OAT initiation prior to and after PDMP implementation.

**Results:**

In total, 1610 people initiated OAT, 946 before and 664 after PDMP implementation. No significant immediate (step) or longer‐term (slope) changes in the rates of OAT initiation were identified following PDMP implementation, after adjusting for seasonality. A high opioid dose (>100 mg oral morphine equivalent) in the 6‐months prior to OAT initiation was the only significant characteristic associated with reduced odds of OAT initiation post‐PDMP implementation (odds ratio 0.29; 0.23–0.37).

**Discussion and Conclusions:**

PDMP implementation did not have a significant impact on OAT initiation among people prescribed opioids. Findings suggest additional clinical initiatives that support OAT initiation are required to ensure PDMPs meet their intended target of reducing opioid‐related harms.

## 1 INTRODUCTION

Opioid prescribing and consumption has increased over the past three decades in countries such as the United States, Canada and Australia [1]. Harms associated with prescription opioids include fatal and non‐fatal overdoses [2, 3], with substantial health, social and economic costs [4, 5]. Long‐term prescription opioid use is associated with increased risk of opioid use disorder (OUD), with opioid dependence among those prescribed long‐term opioids in primary care settings ranging from 3% to 26% [6].

One of the most effective, evidence‐based, clinical treatments for OUD is opioid agonist treatment (OAT) [7]. Recent evidence‐based clinical practice guidelines have suggested avoiding opioid deprescribing for people with severe OUD and instead recommend providing OAT [8]. OAT is associated with a range of improved health outcomes including reductions in all‐cause mortality, overdose mortality and morbidity as well as improved quality of life [9, 10]. Despite this, OAT is often not initiated or treatment is delayed, commonly due to various barriers to accessing treatment, such as stigma and regulatory‐related restrictions and preconceptions [11, 12].

Various policies, adopting demand, supply and harm reduction approaches have been introduced to address opioid‐related harms, including the implementation of prescription drug monitoring programs (PDMP). These programs provide prescribers and pharmacists access to patients' controlled medication history, including the prescribing and dispensing of OAT medications. PDMPs vary between and within countries and outcomes are often inconclusive or contradictory, resulting in both intended and unintended consequences [13, 14, 15, 16, 17]. The majority of PDMP‐related research originates from the United States and focuses on outcomes such as reductions in prescribing, diversion, overdoses and morbidity [14, 15, 18, 19], with few studies exploring the impact of PDMPs in Australia [20, 21]. Less is known about the impact of PDMP implementation on OAT initiation and the role of PDMP in supporting those who have developed OUD to access evidence‐based treatment such as OAT.

Australian jurisdictions have recently introduced a range of opioid‐related policies to mitigate risks and harms associated with prescription opioid use. For example, the state of Victoria implemented its PDMP in April 2019, and was the first Australian jurisdiction to mandate use for community prescribers and pharmacists, which occurred on 1 April 2020. The current study aimed to examine the impact of PDMP implementation on OAT initiation among people prescribed opioids for pain, in Victoria, Australia.

## 2 METHODS

The current retrospective analysis is part of a larger study which is utilising primary care data to explore policy impacts on opioid prescribing and clinical outcomes [22]. The study was approved by the Monash University Human Research Ethics Committee (Number: 24139).

### 2.1 Data source and setting

The POpulation Level Analysis and Reporting (POLAR) general practice analytics platform provided the dataset [23]. Data were sourced from all 464 participating general practices within the POLAR dataset, which are located in the south‐east of Victoria, and are situated within three of Victoria's Primary Health Networks or health regions; Gippsland, East Melbourne and Southeast Melbourne. Victoria has a total of six Primary Health Networks; the three included in the current study comprise both metropolitan and rural regions and represent half of the Victorian population [24].

Non‐identifiable routinely collected data from electronic health records was extracted. Data were extracted for patients aged ≥16 years at cohort entry (1 January 2017), with at least one opioid prescription indicated for pain, which had been prescribed at POLAR sites between 1 April 2017 and 31 December 2020 [24] and excluded patients with a cancer diagnosis during the study period.

### 2.2 Measures

Patients' age (in 10‐year categories), gender and concession card status were directly identified via the POLAR data. Socioeconomic status was measured using the Socio‐Economic Indexes for Areas in quintiles based on each patient's residential postcode [25]. Accessibility/Remoteness Index of Australia Plus [26] of patients' postcode were used to identify remoteness level, which were further categorised into three levels: (i) major cities; (ii) inner regional areas; and (iii) regional and remote areas. Patients' clinical characteristics such as having pain or mental health diagnoses, were determined using Systemized Nomenclature of Medicine—Clinical Terms codes [22]. To determine patients receiving a high opioid dose, a 90‐day moving average opioid dose was calculated in oral morphine equivalents [27], similar to the approach adopted by algorithms to calculate daily opioid dose in the PDMP [27]. The calculations excluded opioids for OAT or cough suppressants.

The outcome of interest for the current study was OAT initiation among people prescribed opioids for pain. OAT was classified as a prescription of either methadone or buprenorphine in a formulation registered in Australia with an indication of treatment for opioid dependence (see Table S1, Supporting Information). OAT initiation was defined as a new OAT prescription between 1 April 2017 and 31 December 2020, with no previous OAT prescriptions in the year prior to initiation.

### 2.3 Statistical analysis

Monthly rates of OAT initiation were examined per 100,000 people who had been prescribed opioids (referred to as ‘the opioid cohort’) in the same month, as well as per 100,000 people in the whole POLAR primary care cohort (referred to as the ‘POLAR primary care cohort’) in the same month. Interrupted time series analysis was adopted to further examine both immediate (step) changes and longer‐term (slope) changes in OAT initiation after voluntary and mandatory PDMP implementation. In total, we included 45 monthly data points in the regression model; 24 monthly data points before voluntary PDMP implementation (April 2017 to March 2019), 12 monthly data points after voluntary PDMP implementation (April 2019 to March 2020) and 9 monthly data points during mandatory PDMP implementation (April 2020 to December 2020). Seasonality was accounted for by adding harmonic terms to the models. Interrupted time series analysis results are presented as level changes affecting the intercept of the time series and trend changes affecting the slope. To further explore the impact of PDMP on OAT initiation among people prescribed opioids, a binary logistic regression model was used to compare the demographic and clinical characteristics of individuals who initiated OAT prior to PDMP implementation (April 2017 and March 2019) and after implementation (April 2019 and December 2020). The model was adjusted for relevant covariates, including age, gender, socio‐economic status, remoteness level and pain and mental health diagnoses. *p*‐values <0.05 were deemed statistically significant in this study.

## 3 RESULTS

We identified 1610 people who initiated OAT, 946 before PDMP implementation (i.e., between April 2017 and March 2019) and 664 after PDMP implementation (i.e., between April 2019 and December 2020). Among this sample, most were men (59.3%), aged 36–45 years (34.4%) and living in metropolitan Melbourne (85.4%). Pain diagnoses were documented in one in two (51.6%), while two in three had a documented mental health condition (65.1%) (Table 2).

**FIGURE 1:**
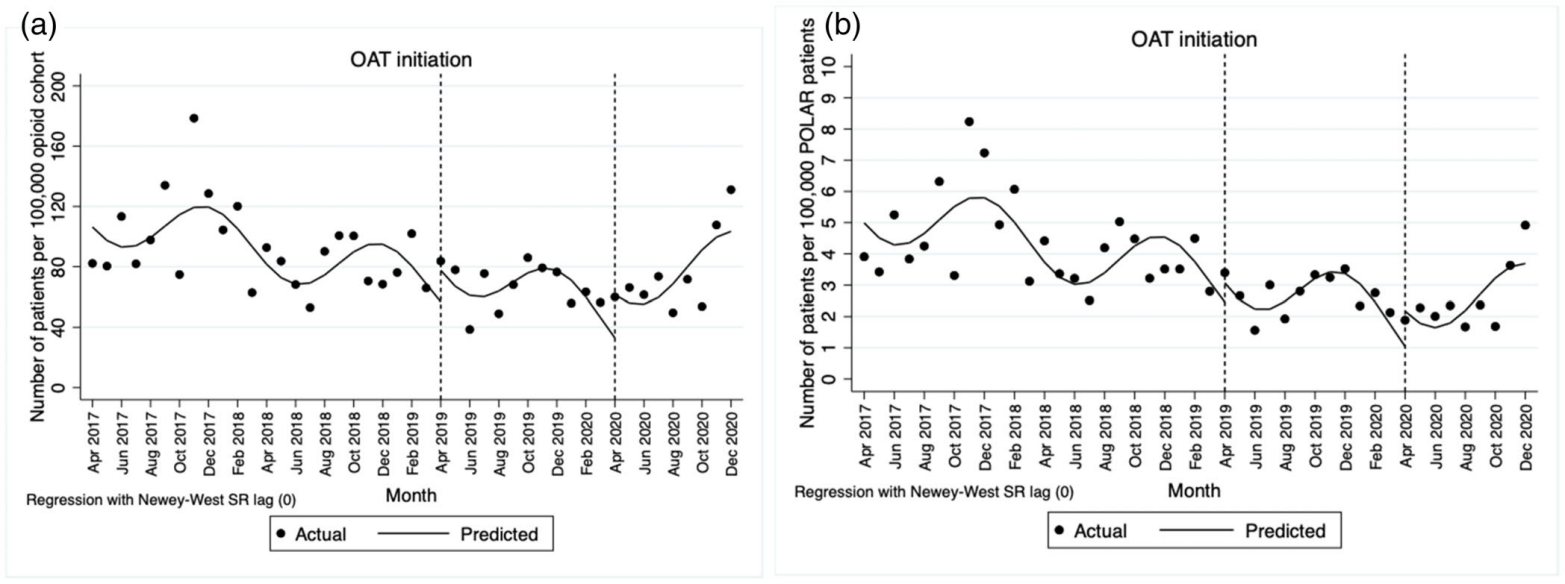
Rates of patients who had initiation of OAT per 100,000 opioid cohort (a) per 100,000 POLAR patients (i.e., the whole primary care cohort, b). Solid line: predicted trend based on the seasonally adjusted interrupted time series model. OAT, opioid agonist treatment.

Table 1 reports the effects of PDMP implementation on rates of OAT initiation per 100,000 in the opioid cohort and the whole POLAR primary care cohort. Prior to PDMP implementation, there was a decreasing rate of OAT initiation among the opioid cohort and the whole POLAR primary care cohort. No significant immediate (step) or longer‐term (slope) changes in the rates of OAT initiation were identified following voluntary (April 2019) and mandatory (April 2020) PDMP implementation, after the adjustment of seasonality (see Figure 1).

**TABLE 1:** Effect of prescription drug monitoring program (PDMP) implementation on rates of OAT initiation per 100,000 opioid cohort and per 100,000 POLAR primary care cohort.

Coefficient	OAT initiation per 100,000 opioid cohort	OAT initiation per 100,000 POLAR primary care cohort
Baseline slope	**−2.05 (−3.60, −0.51)**	**−0.11 (−0.18, −0.03)**
Immediate slope change (Voluntary PDMP—April 2019)	20.69 (−5.66, 47.05)	0.60 (−0.59, 1.79)
Post intervention slope change (Voluntary PDMP—April 2019)	−1.65 (−4.26, 0.95)	−0.06 (−0.17, 0.05)
Immediate slope change (Mandatory PDMP—April 2020)	28.25 (−4.90, 61.40)	1.12 (−0.50, 2.73)
Post intervention slope change (Mandatory PDMP—April 2020)	5.25 (−1.03,11.53)	0.15 (−0.12, 0.43)

*Note*: Bold font denotes significant effects (*p*‐value <0.05).

Abbreviation: OAT, opioid agonist treatment.

Binary logistic regression exploring patient characteristics associated with OAT initiation before and after PDMP implementation revealed that receiving a high opioid dose (>100 mg oral morphine equivalent) in the 6‐months prior to OAT initiation was significantly less likely after PDMP implementation (odds ratio 0.29; 0.23–0.37) (Table 2). No significant difference in gender, age range, concession card status, geographic remoteness, socio‐economic status and having a pain or mental health diagnoses were identified.

**TABLE 2:** Demographic and clinical‐related factors associated with OAT initiation following PDMP implementation (April 2019).

Variables	*N* (%)	Odds ratio
Sex		
Male	955 (59.3)	Reference
Female	655 (40.7)	1.10 (0.89–1.36)
Age		
16–25 years	129 (8.0)	Reference
26–35 years	447 (27.8)	0.74 (0.49–1.13)
36–45 years	554 (34.4)	0.77 (0.51–1.15)
46–55 years	311 (19.3)	0.69 (0.44–1.07)
56–65 years	131 (8.1)	0.82 (0.49–1.37)
66 + years	38 (2.4)	0.56 (0.26–1.23)
Concession card		
Health care and pension concession	1,222 (75.9)	0.92 (0.72–1.18)
Not specified	388 (24.1)	Reference
Remoteness		
Major cities	1,375 (85.4)	Reference
Inner regional	176 (10.9)	1.29 (0.92–1.82)
Outer regional and remote	59 (3.7)	0.98 (0.54–1.76)
Socio‐economic disadvantage		
1‐most disadvantaged	242 (15.0)	Reference
2	162 (10.1)	1.31 (0.85–2.00)
3	402 (25.0)	1.23 (0.87–1.74)
4	365 (22.7)	1.36 (0.95–1.93)
5‐least disadvantaged	439 (27.3)	1.20 (0.85–1.70)
Pain diagnoses		
Not recorded	779 (48.4)	Reference
Recorded	831 (51.6)	0.91 (0.72–1.14)
Mental health conditions		
Not recorded	562 (34.9)	Reference
Recorded	1,048 (65.1)	0.86 (0.68–1.08)
High dose opioid prescribing in 6 months prior OAT		
Not recorded	1,028 (63.9)	Reference
Recorded	582 (36.2)	**0.29 (0.23–0.37)**

*Note*: Bold font denotes significant effects.

Abbreviations: OAT, opioid agonist treatment; PDMP, prescription drug monitoring program.

## 4 DISCUSSION

This is the first Australian study to explore the impact of PDMP implementation on OAT initiation among people prescribed opioids for pain. Results revealed that OAT initiation was decreasing in the lead up to PDMP implementation and this policy did not have a significant impact on rates of OAT initiation. Similar findings were reported in a US study which found mandates to check the PDMP, similar to those adopted in Victoria, had no statistically significant effect on the retail sales of methadone and buprenorphine [28]. PDMP use should help identify patients who would benefit from OAT, however, based on our findings, commencing OAT treatment does not appear to be a common outcome, which suggests a missed opportunity for reducing opioid‐related harms.

Whether OAT was offered and not accepted, or not offered at all is not possible to determine from our study. There are longstanding OAT capacity issues nationally and in Victoria [29], limiting the rate of new OAT entrants more generally [12]. There were no specific resources implemented at the time the PDMP was introduced to help facilitate greater access to OAT, which may have been necessary to alleviate the pressure on an already stretched system to enable more people to access this treatment. Considerable stigma associated with OAT [30], coupled with regulations and restrictions around general practitioners prescribing OAT and an overall reluctance to offer this treatment, are additional barriers to OAT prescribing in general practice [12]. As the prescribing and dispensing of OAT medications are recorded within Victoria's PDMP, it is also possible that other healthcare providers, who have PDMP access (beyond a patient's OAT prescriber), can identify patients receiving OAT more easily. Given the considerable stigma associated with OAT more broadly [30] and possible stigmatising views held by other healthcare providers, this increased visibility may result in increased stigma and further barriers for people on OAT. Conversely, this increased visibility may in fact help to break down stigma and barriers and therefore future research exploring this complex phenomenon is warranted, to help avoid possible unintended consequences, of opioid policies such as PDMPs.

The impact of stringent COVID‐19 lockdown restrictions in Victoria, placing pressure on healthcare services, may have also potentially contributed to reduced OAT initiation [31]. Time pressures and limited interactions with patients while using telehealth may have further impeded opportunities for prescribers to identify patients who may benefit from OAT. Victoria's PDMP implementation did not appear to have meaningful impacts on high‐dose or high‐risk combination medication prescribing, which is consistent with this as a potential explanation [20].

While several other studies have reported demographic (e.g., age, gender, ethnicity) and clinical characteristics (e.g., mental health diagnosis, prescribed medications and doses, OAT type and dose) associated with OAT initiation [32] and retention [33, 34], less is known about characteristics associated with OAT initiation following PDMP implementation. A high opioid dose in the 6 months prior to OAT initiation was the only significant characteristic associated with reduced odds of OAT initiation following PDMP implementation. OUD is often associated with both long‐term and high‐dose opioid use [35, 36, 37]. Despite no overall impact of PDMP on high‐dose prescribing [20], this current study may have identified a subpopulation who transitioned to lower opioid doses post‐PDMP implementation, or this could reflect a general background trend of declining opioid doses.

It is also possible that while the PDMP is designed to identify medication‐related risks, through an algorithm which triggers high risk alerts, it does not provide a clinical support system to assist prescribers with how best to respond to these alerts, including the processes and supports to initiate OAT. It may therefore be possible that existing system barriers coupled with a possible reluctance to transfer patients on high doses to OAT, may also explain the current finding. Further research exploring why there are reduced odds of OAT initiation following PDMP implementation are therefore needed. In general, it appears that the characteristics of those seeking OAT following PDMP implementation are largely similar to the characteristics of those who were seeking it prior, with the exception of fewer being on high opioid doses.

### 4.1 Strengths and limitations

The current study utilises data from one of Australia's largest primary care research datasets and is the first Australian study to explore the impact of PDMP implementation on OAT initiation, among people prescribed opioids. The following limitations should be considered when interpreting the results. The current study aimed to determine the impact of PDMP implementation on OAT initiation among people prescribed opioids for pain, and therefore may not be representative of all OAT patients, including those who use non‐prescribed opioids. Future research to determine the impact of PDMP implementation among a broader sample that includes people who use heroin is warranted. Mandatory PDMP use coincided with Victoria's first COVID‐19 restrictions, which included reduced face‐to‐face medical consultations. These restrictions may have impacted OAT initiation, despite strategies in place to support access to OAT more generally for those already on OAT, during the pandemic [38, 39]. POLAR data was collected at a practice level rather than patient level; therefore it is possible patients may be attending multiple general practices. However, patients receiving OAT and/or long‐term opioids (>8 weeks) are restricted to one provider through a permit system, and therefore it is unlikely this would impact the results significantly. Patients may have initiated OAT outside of general practice and would not be included in the POLAR dataset, however, in Victoria most (>95% of people) access OAT through primary care [40]. Finally, we only examined data for 8‐months after mandatory implementation, making it important to conduct further studies looking at longer‐term outcomes.

## 5 CONCLUSION

Implementation of PDMP did not have a significant impact on declining rates of OAT initiation among people prescribed opioid for pain, in Victoria, Australia. It appears this is a missed opportunity for prescribers to identify patients with OUD who may benefit from OAT. These findings suggest further clinical initiatives are required when implementing PDMPs, to ensure they meet their desired impact on reducing opioid‐related harms. Future studies exploring OAT initiation over longer periods of time and across other Australian jurisdictions following PDMP implementation are warranted.

## AUTHOR CONTRIBUTIONS

SN conceptualised and received funding for the study with chief and associate investigators (JSB, CP, RB and DL). TX performed the analyses. The original draft was produced by LP. All authors were involved in manuscript revision and jointly take responsibility for all aspects of the work.

## CONFLICT OF INTEREST STATEMENT

Louisa Picco, Ting Xia, Christopher Pearce and Rachelle Buchbinder report no conflicts. Suzanne Nielsen has received untied educational grants to study pharmaceutical opioid related harm from Seqirus, and is a named investigator on an implementation trial of buprenorphine depot funded by Indivior (no funding received by SN personally or through her institution), both unrelated to this work. Dan I. Lubman has received funding for investigator sponsored studies related to depot buprenorphine from Camurus, unrelated to this work. J. Simon Bell has received grant funding or consulting funds GlaxoSmithKline Supported Studies Programme, Amgen, and several aged care provider organisations unrelated to this work. All grants and consulting funds were paid to the employing institution.

## Supporting information


**Table S1:** Opioid agonist treatment formulations in Victoria, Australia*.
