# Supplementary material for: Changes in opioid agonist treatment initiation among people prescribed opioids for pain following voluntary and mandatory prescription drug monitoring program implementation: A time series analysis
Source: Drug Alcohol Rev. 2023 Oct 5;42(7):1639–46. doi: 10.1111/dar.13754 (PMC10947011; doi:10.1111/dar.13754)
Supplement: Supplementary file 1 — Table S1: Opioid agonist treatment formulations in Victoria, Australia*. [file DAR-42-1639-s001.docx]

**Table S1. Opioid agonist treatment formulations in Victoria, Australia***

| Medicines for OAT | Formulation/strength/brand name |
| --- | --- |
| Buprenorphine | - Buprenorphine sublingual tablet 0.4 mg, 2 mg, 8 mg (Subutex®) - Buprenorphine/naloxone sublingual film 2 mg/0.5 mg, 8 mg/2 mg (Suboxone®) - Buprenorphine injections^ (Buvidal® Weekly, Buvidal® Monthly or Sublocade®) *various strengths* |
| Methadone | - Methadone hydrochloride oral syrup 5mg/mL (Aspen Methadone® or Biodone Forte®) |

*OAT programs vary between Australian jurisdictions including the OAT medications most commonly used, regulatory requirements and the settings and services in which OAT is provided. In Victoria, OAT predominantly comprises two medicines, methadone and buprenorphine. A permit system is required whereby prescribers and pharmacists are required to complete a registered training and subsequently be granted permission by the Department of Health and Human Services to supply OAT.

**^** Buprenorphine injections were included but were not found in the dataset

OAT, opioid agonist treatment.
